# Liver mitochondrial function in ZDF rats during the early stages of diabetes disease

**DOI:** 10.14814/phy2.12686

**Published:** 2016-02-04

**Authors:** Guillaume Vial, Marie Le Guen, Frédéric Lamarche, Dominique Detaille, Cécile Cottet‐Rousselle, Luc Demaison, Isabelle Hininger‐Favier, Pierre Theurey, David Crouzier, Jean‐Claude Debouzy, Hervé Dubouchaud, Éric Fontaine

**Affiliations:** ^1^ Facultés de médecine Charles Mérieux Lyon‐Sud et RockfellerINSERM U‐1060Laboratoire CarMeNUniversité Lyon 1, INRA 1235, INSA de LyonLyonFrance; ^2^ European Center For Nutrition and HealthCentre Hospitalier Lyon SudPierre‐BéniteFrance; ^3^ Laboratoire de Bioénergétique Fondamentale et Appliquée (LBFA) et SFR Biologie Environnementale et Systémique (BEeSy)INSERM U‐1055GrenobleFrance; ^4^ Joseph Fourier UniversityGrenobleFrance; ^5^ Grenoble University HospitalGrenobleFrance; ^6^ Centre de Recherche Cardio‐Thoracique de Bordeaux – CRCTB – LIRYC Université de Bordeaux 2, BordeauxINSERM U‐1045Hôpital Xavier Arnozan, BordeauxBordeauxFrance; ^7^ Unité de Nutrition HumaineINRA, UMR 1019Clermont UniversitéUniversité d'AuvergneClermont‐FerrandFrance; ^8^ Institut de Recherche Biomédicale des Armées‐Unité des Risques Technologiques EmergentsBrétigny sur OrgeFrance

**Keywords:** Diabetes, mitochondria, oxidative phosphorylation, ZDF

## Abstract

The aim of this study was to characterize the early alterations of the liver mitochondrial function in ZDF (fa/fa) rats that develop diabetes compared to that of their lean counterparts ZDF (fa/+). Liver mitochondrial function was examined in 11‐ and 14‐week‐old ZDF (fa/fa) and ZDF lean (fa/+) rats. Oxygen consumption, H_2_O_2_ release, calcium retention capacity (CRC), membrane potential, membrane fluidity, and fatty acid composition were analyzed. State 3 oxygen consumption with palmitoyl‐carnitine increases between 11 and 14 weeks of age in lean but not in diabetic animals. This response was not seen with other substrates, suggesting that the use of fatty acids is impaired in diabetic rats. H_2_O_2_ release was lower in 14‐week‐old ZDF (fa/fa) rats as compared to ZDF lean (fa/+). These changes were not associated with differences in enzymatic activities of the respiratory complexes, suggesting regulatory mechanisms independent of their expression levels. Membrane fluidity and composition analyses show only slight effects linked to diabetes progression. The most salient feature was a reduction in CRC in the presence of CsA, an effect reflecting PTP dysregulation. Our data suggest few changes of mitochondrial function in ZDF fa/fa rats. At the age of 11 weeks, liver mitochondria have mainly a reduced effect of CsA on CRC.

## Introduction

Factors underlying the establishment of type 2 diabetes (T2D) have not been fully elucidated despite a large number of investigations in the field; however, the relationship between insulin resistance, hyperglycemia and mitochondrial function is increasingly gaining interest and remains a new therapeutic approach (Lowell and Shulman 2005; Vial et al. 2010, 2015; Hoeks and Schrauwen 2012; Rocha et al. 2013). Many studies have focused on skeletal muscle mitochondria. The majority of such studies suggests a decrease in oxidative capacity leading to an accumulation of diacylglycerol and an increase in ROS production responsible for the disruption of insulin signaling in T2D patients (Kelley et al. 2002; Mootha et al. 2003; Patti et al. 2003; Petersen et al. 2003, 2004; Morino et al. 2005; De Feyter et al. 2008; Szendroedi et al. 2014; Toledo 2014). A similar mechanism has been shown to take place in the liver but is less described (Petersen and Shulman 2006; Erion and Shulman 2010; Magkos et al. 2012; Martin and McGee 2014; Perry et al. 2014). Moreover, very few studies have been done on liver mitochondria isolated from the well‐known insulin‐resistant rat model ZDF fa/fa used in pharma research. Brady et al. have shown on a small group of this animal model that mitochondrial capacity to oxidize fatty acids is not altered (Brady and Hoppel 1983). More than 10 years later, Ramsey et al. presented a study reporting an increase in mitochondrial proton leak in ZDF obese rat (Ramsey et al. 1996). However, a large study performed by Brookes demonstrated that the proton leak at 37°C was similar in obese and control Zucker rats, causing doubt (Brookes et al. 1998). Recently, these results were reinforced as there is no dysfunction in liver mitochondria extracted from ZDF rats at the age of 11 weeks (Flamment et al. 2008). We noticed that the age of animals used was very close after the transition of the prediabetic state into full‐blown T2D. Considering this, we hypothesized that mitochondrial dysfunction could appear later as a consequence of hyperglycemia. In this study, we used ZDF (fa/fa) rats at the age of 11 weeks (3 weeks after the increase in blood glucose) and 14 weeks (6 weeks after the increase in blood glucose) and their control littermates (ZDF lean with normal glycemia) (fa/+) to clarify the impact of hyperglycemia on mitochondria with appropriate controls as it rarely have been done before (Brady and Hoppel 1983; Ramsey et al. 1996; Brookes et al. 1998; Flamment et al. 2008). We examined mitochondrial respiration, H_2_O_2_ release and calcium retention capacity (CRC) as well as mitochondrial membrane potential, composition, and specifications to determine whether mitochondrial dysfunction is involved in the early events after establishment of hyperglycemia.

## Methods and Procedures

### Animals and experimental design

Seven‐week‐old male ZDF fa/fa rats (obese) (*n* = 14) or ZDF lean (fa/+) (*n* = 14) were housed individually at 22°C, in 12 h/12 h light/dark conditions and with a 50% relative humidity. Rats were randomly assigned to be euthanized at 11 weeks of age (11w group, *n* = 7) or at 14 weeks of age (14w group, *n* = 7). Body weight and food intake were recorded twice a week. The experiments followed the European Union recommendations concerning the care and use of laboratory animals for experimental and scientific purposes. All animal procedures have been approved by the Comité Régional d'Ethique pour l'Expérimentation Animale Rhone‐Alpes (regional board of ethics) for advice and was notified to the research animal facility of the Department of Biology, Université Joseph Fourier Grenoble.

### Mitochondrial oxygen consumption and ROS release

Rat liver mitochondria were isolated according to a standard differential centrifugation procedure in 250 mmol L^−1^ sucrose, 20 mmol L^−1^ Tris‐HCl, 1 mmol L^−1^ EGTA, pH 7.4 (Klingenberg and Slenczka 1959). Protein concentrations were determined using the bicinchoninic acid assay using BSA as a standard (Pierce). The rate of mitochondrial oxygen consumption (*J*O_2_) was measured at 30°C using a Clark‐type O_2_ electrode in a 1 mL‐chamber filled with respiration buffer: 125 mmol L^−1^ KCl, 10 mmol L^−1^ Pi‐Tris, 20 mmol L^−1^ Tris‐HCl, 1 mmol L^−1^ EGTA, 0.15% FFA‐BSA, pH 7.2 and using 1 mg of mitochondrial proteins mL^−1^. Measurements were carried out in the presence of either glutamate (5 mmol L^−1^)/malate (2.5 mmol L^−1^) and/or succinate (5 mmol L^−1^) or palmitoyl‐carnitine (55 μmol L^−1^) as substrates (state 2), after the addition of 1 mmol L^−1^ ADP (state 3), followed by the addition of 0.25 mg mL^−1^ oligomycin (state 4). ROS release was estimated by measuring H_2_O_2_ release in a stirred 1 mL‐chamber containing 0.2 mg of mitochondria and filled with a respiration buffer containing 6 UI horseradish peroxidase and 1 μmol L^−1^ Amplex Red^®^ (excitation: 560 nm; emission: 584 nm) and the same substrates as for respiration. Measurements were carried out both in basal conditions and after sequential additions of 2 μmol L^−1^ rotenone and 2 μmol L^−1^ antimycin A. Results were expressed in pmol H_2_O_2_ min^−1^ mg Prot^−1^ using H_2_O_2_ standard solutions as we have already described (Vial et al. 2011).

### Mitochondrial calcium retention capacity (CRC)

Mitochondria (1 mg mL^−1^) were incubated at 30°C in 1 mL of buffer (250 mmol L^−1^ sucrose, 10 mmol L^−1^ Mops, 1 mmol L^−1^ Pi‐Tris, 0.15% FFA‐BSA, pH 7.4) containing glutamate–malate (5–2.5 mmol L^−1^) or succinate–rotenone (5 mmol L^−1^–2.5 μmol L^−1^). Changes in extramitochondrial calcium concentration were monitored fluorimetrically (Hitachi, F4500 spectrofluorometer) using 0.25 μmol L^−1^ Calcium Green‐5N (excitation: 506, emission: 530 nm) as described by Ichas et al. (Ichas et al. 1997). Unless stated otherwise, calcium pulses (25 μmol L^−1^) were then added at 1 min intervals until a Ca^2+^‐induced mitochondrial Ca^2+^ release was observed. CRC was taken as the total amount of Ca^2+^ accumulated by mitochondria prior to the Ca^2+^ pulse triggering Ca^2+^ release. This value represents a reliable index of the threshold Ca^2+^ concentration required to open the permeability transition pore (PTP) in the whole mitochondrial population studied (Fontaine et al. 1998).

### Determination of mitochondrial membrane potential by flow cytometry

Isolated mitochondria (1 mg mL^−1^) were incubated with different substrates (same conditions as for measurement of ROS production) and TMRM (100 nmol L^−1^) for 10 min at 30°C under slight agitation in 5 mL flow cytometer polypropylene tubes containing KCl Buffer supplemented with 0.15% BSA and 1 mmol L^−1^ Pi‐Tris. Total fluorescence was determined and then 50 μmol L^−1^ CCCP (uncoupling agent) was added and samples were reanalyzed. Results were normalized as a ratio between total TMRM fluorescence and TMRM fluorescence in the presence of CCCP, that is, in uncoupling state. Nonlabeled mitochondria were used as autofluorescence control to adjust instrument settings. Calibrated beads were used to control laser stability for each experiment. The TMRM fluorescence was obtained after excitation by a 150 mW argon ion laser tuned at 532 nm and collected with a 585/15 nm band‐pass filter. Data acquisition (5000 events) was carried out in a LSR^TM^ FORTESSA SORP flow cytometer (Becton Dickinson Biosciences, Le Pont‐de‐Claix, France), using the FACSDiVa^TM^ software (Becton‐Dickinson Biosciences) (Vial et al. 2011).

### Mitochondrial membrane fluidity assayed by ESR experiments

Effect of diet on mitochondrial membrane fluidity was assessed by ESR spin label experiments. Two spin labels (Sigma France) were used: 5‐nitroxide stearate (5 NS) and 16‐nitroxide stearate (16 NS). These fatty acids self‐incorporate the membrane and the nitroxide groups provide information of motional freedom of the label in the system. So 5NS probes the superficial part of the membrane layer, whereas 16 NS probes its hydrophobic core (Debouzy et al. 2007).

The experiments performed on liver mitochondria. 100 *μ*L of mitochondria suspension was labeled with 2.5 *μ*L of spin label solution (50 mmol L^−1^ 5 NS or 5 mmol L^−1^ 16 NS). The ESR spectra were recorded at different controlled temperature (294, 295, 296, 297, 298, and 299°K) with the following conditions: microwave power 10.00 mW, modulation frequency 100 kHz, modulation amplitude 1.03 G, receiver gain 1 × 10^5^ conversion time 20.48 msec, time constant 20.48 msec. Sweep range was 100 G with a central field value of 3435 G for 5 NS probe, and in the same condition except, modulation amplitude 0.51 G, receiver gain 6.3 × 10^5^ conversion time 20.48 msec, time constant 20.48 msec for 16 NS probe.

The complete membrane incorporation of the spin labels was ascertained by the absence on the spectra of the extremely resolved ESR lines corresponding to free rotating markers.

5 NS experimentations: The values of outer and inner hyperfine splitting were measured (2*T* **//**and 2*T*⊥, respectively), on ESR spectra, and order parameter *S* was calculated following the equation (Petersen et al. 2003):

$$S=1.723\times \frac{T//-(T⊥+C)}{T//+2(T⊥+C)}$$

with C=1.4−0.053×(T//−T⊥)

The increase in the order parameter value means a decrease in local membrane fluidity.

16 NS experimentations: The changes in freedom motion of 16 NS were analyzed with the calculation of *τ*_c,_ the rotational correlation time. *τ*_c_ was calculated following the formula (Toledo 2014):

$$T_{c}=K\times \Delta W_{0}\left(\sqrt{(h_{0}/h_{-1}}-1\right)$$

with $K=6.5\times 10^{-10}s.G^{-1}$

In this formula, Δ*W*_0_ is the peak‐to‐peak line width of the central line; *h*_0_ and *h*_−1_ are the peak heights of the central and high‐field lines, respectively.

The increase in the rotational correlation time means a decrease in local membrane fluidity.

### Mitochondrial membrane specifications assayed by liquid NMR

Total lipid extracts were realized following the method of Bligh and Dyer by using chloroform/methanol/water 2/1/0.8 mixture (Vol/Vol/Vol) (Bligh and Dyer 1959). The lipid phase was evaporated and resuspended in 500 *μ*L CDCl_3_/MeOD 4/1 (Vol/Vol) to allow field lock.

The resonances were attributed as labeled below for a typical model chain:

CH_3_‐CH_2_‐(CH_2_)n‐CH_2_‐CH=CH‐CH_2_‐CH=CH‐CH_2_‐(CH2)n‐CH_2_‐CH_2_‐CO‐7 6 6 5 4 4 3 4 4 5 6 2 1

Terminal methyl of acyl chains 7 (resonance at 0.8 ppm) and of *ω*3 bearing chains (peak labeled W at 0.85 ppm); Methylenic resonances, 6 (1.2 ppm); 5 (1.95 ppm); 3 (2.7 ppm); 2 (1.4 ppm); 1 (2.2 ppm); Methynic resonances: 4 (5.4 ppm), and Glycerol‐beta, G (5.1 ppm).

The integrals on these resonances were then used to build several index representative of: Chain length (6 + 5+4 + 3+2 + 1)/(7 + W); Chain unsaturation: 4/(7 + W + 6 + 5+4 + 2+1); Chain polyunsaturation: 3/(7 + W + 6 + 5+4 + 2+1); Relative contribution of *ω*3: W/(W + 7); Chain to glycerol ratio: (7 + W + 6 + 5+4 + 3+2 + 1)/G.

All high‐resolution RMN spectra in liquid were recorded on a Bruker AM400 spectrometer (9.4T) operating at 400.13 MHz for proton.

^1^H‐NMR spectra were acquired on a Bruker AVANCE 400 spectrometer (9.4T); recycling delay was 1 sec. 32K data points were collected on a spectral width of 10 ppm. 256 scans were used for each spectrum. A line‐broadening of 0.2 Hz was also applied before Fourier Transform. The attributions of the resonances were drawn from the literature and from classical NMR methods (COSY, TOCSY).

### Liver oxidative stress

Five hundred milligram of tissue sample were extracted in buffer (10 mmol L^−1^ Tris‐NaOH, 1 mmol L^−1^ DPTA, 1 mmol L^−1^ PMSF, pH = 7.4) and centrifuged at 3000 g and 4°C for 10 min.

Lipid peroxidation was assessed by measuring the concentration of thiobarbituric acid reactive substances (TBARS) in liver homogenates as described by Richard et al. (Richard et al. 1992), using a fluorimetric determination of malondialdehyde (MDA)‐TBA (thiobarbituric acid) complex after extraction with n‐butanol. The concentration of liver SH protein groups was determined using 5′‐dithiobis (2‐nitrobenzoic acid) to derivative SH groups which were subsequently measured at 412 nm as already described (Hininger et al. 2005). Liver Se‐GPx activity was evaluated by the method of Gunzler using terbutyl hydroperoxide as substrate instead of hydroperoxide. Results were expressed as nmoles of NADPH oxidized per minute as unit per liter. Total glutathione was determined by the modified method of Akerboom (Akerboom and Sies 1981).

The ratio between the activities of aconitase and fumarase of the liver was calculated as an indicator of mitochondrial ROS production damage (Gardner et al. 1994). Mitochondrial aconitase is sensitive to inactivation by superoxide due to the susceptibility of its iron‐sulfur core to oxidation while fumarase is unaffected. Aconitase and fumarase activities were determined according to Gardner et al. (Gardner et al. 1994), but were measured after extraction with a medium supplemented with citrate sodium (1 mol L^−1^) in order to stabilize the aconitase activity ex vivo. Values of aconitase and fumarase activities were determined on the same extract for each biological sample.

### Western blot analysis

Mitochondria were lysed in PBS containing 1% NP‐40, 0.5% sodium deoxycholate, 0.1% SDS supplemented with 5 mmol L^−1^ EDTA, 1 mmol L^−1^ Na_3_VO_4_, 20 mmol L^−1^ NaF, 1 mmol L^−1^ DTT, and protease inhibitor cocktail (Sigma P2714). Proteins were separated by SDS‐10% PAGE, transferred to polyvinylidene difluoride (PVDF) membrane, and incubated overnight with primary antibodies. Primary antibodies used were OXPHOS (Abcam, ab110413), ANT2 (Santa Cruz, SC‐9299), UCP2 (BioLegend, 615902), and actine (Sigma A5060).

For cyclophilinD (CypD: Ppif) content determination, samples were frozen and heated five times and then centrifuged at 100,000 × *g* for 3 h. Supernatant were collected and considered as the matrix content. Pellets were washed with PBS, centrifuged at 100,000 × *g* for 3 h and proteins were extracted with PBS containing 1% NP‐40, 0.5% sodium deoxycholate, 0.1% SDS supplemented with 5 mmol L^−1^ EDTA, 1 mmol L^−1^ Na_3_VO_4_, 20 mmol L^−1^ NaF, 1 mmol L^−1^ DTT, and protease inhibitor cocktail (Sigma P2714). 12 *μ*g of proteins was separated by SDS‐15% PAGE, transferred to polyvinylidene difluoride (PVDF) membrane, and incubated overnight with primary CypD antibodies (Abcam, ab110324). Signals were detected with a horseradish peroxidase‐conjugated secondary antibody (Biorad, 172‐10‐19) and revealed with an enhanced chemiluminescence system (Pierce).

### Statistical procedures

All data are presented as mean ± SE. Two‐way analysis of variance (ANOVA) was used to determine the global effect of age and genotype. When appropriate, differences between groups were tested with a PLSD Fisher post hoc test. Statistical significance was accepted at *P *<* *0.05. Mann–Whitney tests were applied when values were non‐normally distributed.

## Results

### Animal characteristics

ZDF fa/fa rats present higher body weight and blood glucose levels starting at the age of 8 weeks compared to their lean counterparts. At the age of 11 weeks, rats were characteristically hyperglycemic as shown by significantly elevated blood glucose and glycosylated hemoglobin levels compared to their own littermate lean animals as indicated in Table 1. In ZDF fa/fa rats, elevated blood glucose is associated with hyper‐insulinemia at 11 and 14 weeks of age (Table 1). Finally, ZDF fa/fa 11w rats can be considered as diabetics less than 3 weeks, whereas ZDF fa/fa 14w rats can be considered as diabetics for 6 weeks.

**Table 1:** Animal's characteristics

	8 weeks		11 weeks		14 weeks	
	lean	fa/fa	lean	fa/fa	lean	fa/fa
Body weight (g)	207 ± 7	228 ± 6^a^	298 ± 4	360 ± 4^a^	314 ± 6	365 ± 9^a^
Liver (% body weight)	ND	ND	3.31 ± 0.05	4.73 ± 0.09^a^	3.23 ± 0.05	4.92 ± 0.10^a^
Gastrocnemius (% body weight)	ND	ND	0.47 ± 0.01	0.28 ± 0.01^a^	0.45 ± 0.01	0.27 ± 0.01^a^
Abdominal adipose tissue (% body weight)	ND	ND	1.28 ± 0.06	3.54 ± 0.09^a^	1.46 ± 0.04	3.87 ± 0.11^a^
Food intake (g/day)	ND	ND	18.5 ± 1.5	37 ± 8^a^	18.7 ± 1.2	34 ± 5^a^
Blood glucose (mg dL^−1^)	115 ± 3	136 ± 9	111 ± 3	480 ± 19^a^	112 ± 3	479 ± 19^a^
HbA1C (%)	4.1 ± 0.03	4.2 ± 0.09	4.12 ± 0.06	8.86 ± 0.6^a^	4.16 ± 0.1	10.76 ± 0.6^a^
Insulin (ng mL^−1^)	ND	ND	1.32 ± 0.15	4.54 ± 0.76^a^	1.68 ± 0.24	4.18 ± 0.57^a^

Data are presented as mean ± SEM (*n* = 6). ND, not determined.

a Significantly different than lean (*P* < 0.05) in each row.

### Mitochondrial oxygen consumption

**Figure 1:**
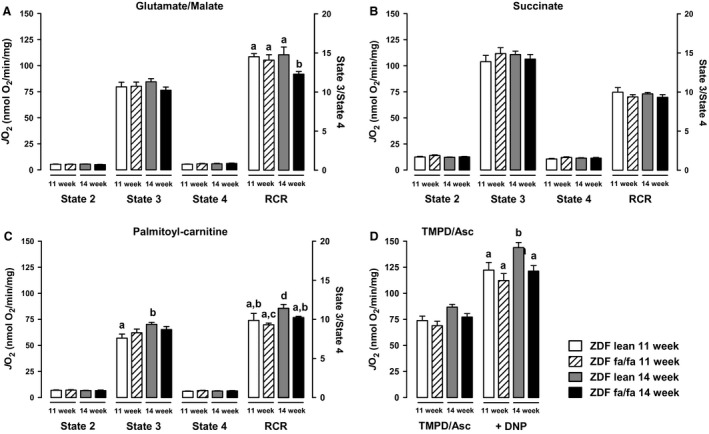
ZDF fa/fa or lean liver mitochondrial oxidative capacities. Liver mitochondria were isolated as described in Materials and Methods. Respiratory rate was determined at 30°C by incubating mitochondria (1 mg mL ^−1^) with glutamate/malate (GM) (A), succinate (B), Palmitoyl‐carnitine (C), without ADP (state‐2), in the presence of 1 mmol L^−1^ ADP (state‐3) or 5 *μ*g mL ^−1^ oligomycin A (state‐4) or with TMPD‐Asc (D) + DNP. Values are expressed as means ± SEM (*n* = 7 rats in each group). *a* ≠ *b* with *P* < 0.05 on each graph.

First, we investigated whether genotype and age, that is, the duration of diabetes, affect mitochondrial respiration by assessing the respiration rate of freshly isolated liver mitochondria with either glutamate/malate, succinate, palmitoyl‐carnitine, or TMPD/Ascorbate as substrates. Mitochondrial state‐3 (phosphorylating) oxygen consumption was identical in ZDF fa/fa rats compared to ZDF lean, when glutamate/malate or succinate substrates were used (Fig. 1A and B). In contrast, state‐3 oxygen consumption in the presence of palmitoyl‐carnitine as well as under uncoupled condition TMPD/ASc and DNP, significantly increased (respectively, +23%, *P* < 0.05 and +18%, *P* < 0.05) from 11 to 14 weeks of age in ZDF lean but not in diabetic animals (Fig. 1C and D). State‐2 and state‐4 respiration rates were not modified by the genotype and the duration of diabetes whatever the substrate used. Respiratory control ratios calculation (RCR: state‐3/state‐4) with G/M (14.5 ± 0.4 and 14.1 ± 0.7 in 11‐week‐old lean rats vs. fa/fa, 14.8 ± 0.9 and 12.3 ± 0.3 in 14‐week‐old lean rats vs. fa/fa) indicates high‐quality mitochondria and efficient oxidative phosphorylation coupling. Notwithstanding, ZDF fa/fa 14w mitochondria presented a significant decreased of RCR compared to ZDF lean 14w (*P* = 0.019). Similar observations were showed with palmitoyl‐carnitine, but were less marked with succinate.

### Mitochondrial ROS release and damage

**Figure 2:**
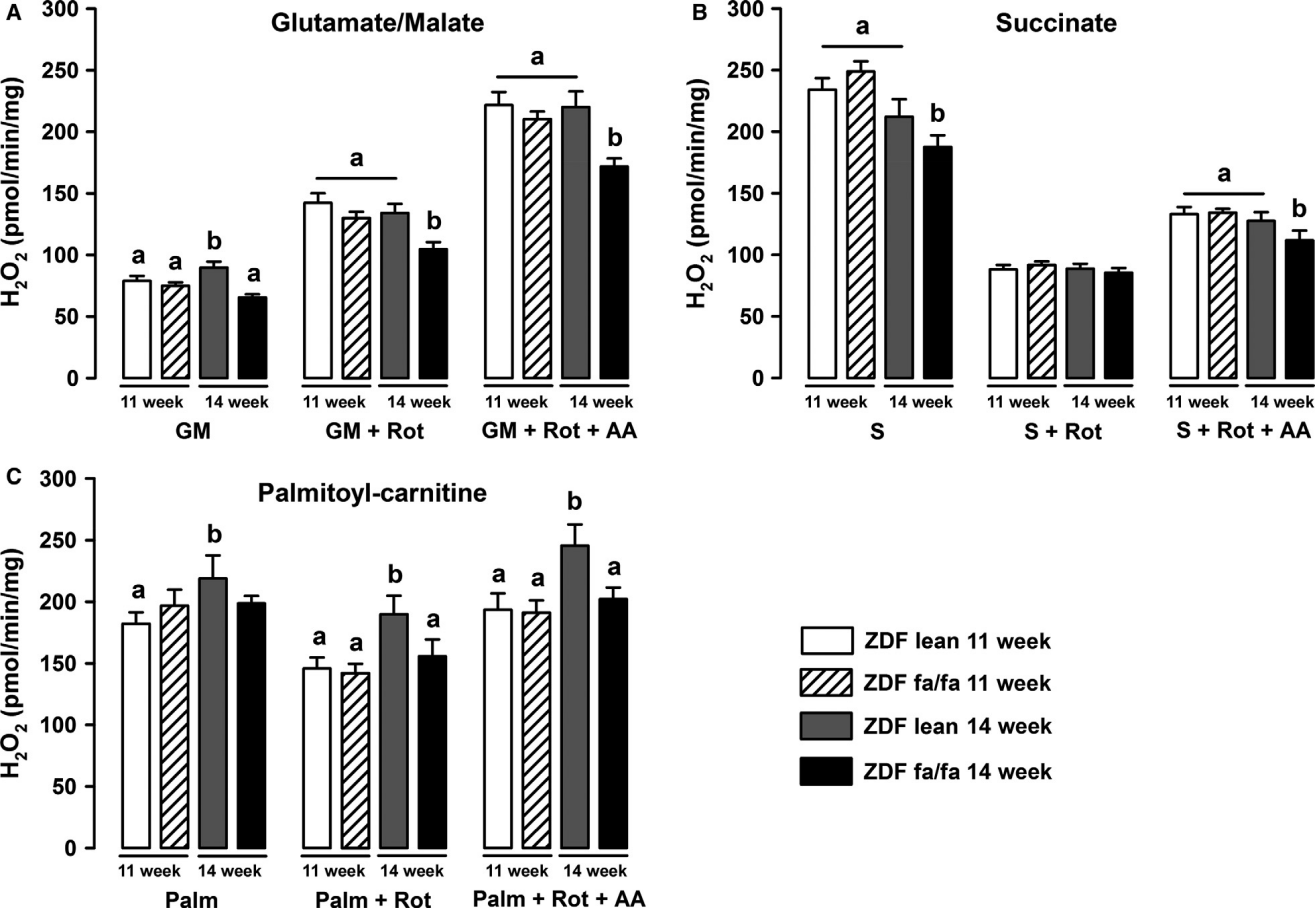
ZDF fa/fa or lean liver mitochondrial H_2_O_2_ production. Liver mitochondria were isolated as described in Materials and Methods. H_2_O_2_ production rate was determined at 30°C by incubating mitochondria (0.2 mg mL ^−1^) in a respiration buffer (see Materials and Methods) with 6 IU horseradish peroxidase and 1 μmol L^−1^ Amplex Red. Measurements were carried out with various substrates: 5 mmol L^−1^ glutamate/2.5 mmol L^−1^ malate (A), 5 mmol L^−1^ succinate (B), or Palmitoyl‐carinitine (C) and after sequential addition of rotenone (Rot) and antimycin A (+Rot+AA). Values are expressed as means ± SEM (*n* = 7 rats in each group). *a* ≠ *b* with *P* < 0.05 on each graph.

Liver mitochondria from ZDF lean rats displayed an increase in H_2_O_2_ basal production with age when glutamate/malate or palmitoyl‐carnitine were used as substrates (Fig. 2). Basal mitochondrial H_2_O_2_ release with succinate remained stable in ZDF lean rats in contrast to ZDF fa/fa mitochondria in which it decreased with age (Fig. 2B, – 25% vs. ZDF fa/fa 11w, *P* < 0.05). In conditions established to performed reverse electron flux through complex I, that is, in the presence of succinate (compare to succinate + rotenone), H_2_O_2_ release by respiratory chain was lower in ZDF fa/fa 14w rats as compared to ZDF fa/fa 11w and lean rats. H_2_O_2_ productions after addition of antimycin A subsequently to rotenone, with G/M or succinate as substrates were still lower in ZDF fa/fa 14w rats.

Otherwise, liver GPx activity was elevated in ZDF fa/fa rats. However, GSH red concentration was lower in these animals. Aconitase to fumarase ratio was higher in ZDF fa/fa rats at the age of 11 weeks, whereas it was identical to the ratio of ZDF lean at 14 weeks (Table 2).

**Table 2:** Liver oxidative stress

	11 weeks		14 weeks	
	lean	fa/fa	lean	fa/fa
GPx (U g Prot^−1^)	1562 ± 70^a^	1829 ± 31^b^	1681 ± 49^a^	1867 ± 46^b^
Thiols (*μ*mol g Prot^−1^)	113 ± 3^a^	105 ± 4	110 ± 5^a^	95 ± 3^b^
Frap (*μ*mol g Prot^−1^)	98 ± 3	105 ± 5	112 ± 2	101 ± 5
GSH tot (μmol L^−1^)	1567 ± 60	1433 ± 51	1593 ± 41	1443 ± 22
GSSG (μmol L^−1^)	10 ± 1^a^	11 ± 1^a^	17 ± 3^b^	16 ± 2^b^
GSH red (μmol L^−1^)	1548 ± 60^a^	1411 ± 50^b^	1560 ± 39^a^	1410 ± 22^b^
Aconitase/Fumarase	5.80 ± 0.32^b^	7.68 ± 0.93^a^	6.03 ± 0.56^a,b^	5.31 ± 0.50^b^

Liver GPx activity, Thiols, and Frap as well as glutathione total, oxidized and reduced were assessed in liver of ZDF rats. The ratio between aconitase to fumarase activities was calculated as an indicator of ROS production damage. Data are presented as mean ± SEM (*n* = 7). *a* ≠ *b* with *P* < 0.05 on each line. a and b are statistically different.

### Mitochondrial membrane potential, composition, and fluidity

**Figure 3:**
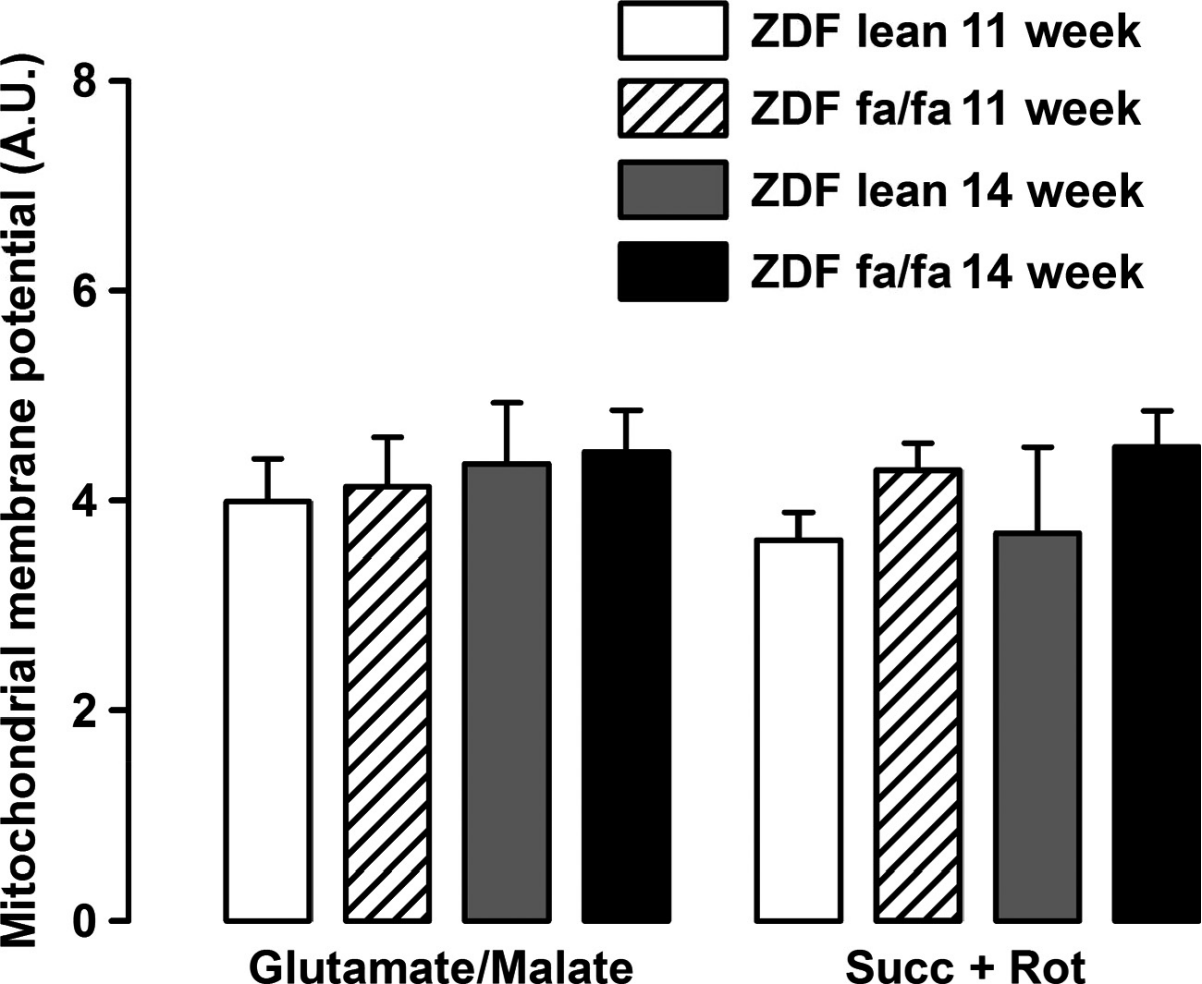
ZDF fa/fa or lean mitochondrial membrane potential. Liver mitochondria were isolated as described in Materials and Methods. Mitochondria were incubated in same conditions used to measured H_2_O_2_ production and were labeled with TMRM. Fluorescence were analyzed from 5000 events and normalized to the fluorescence in the presence of CCCP. Values are expressed as means ± SEM (*n* = 7 rats in each group).

The use of TMRM fluorescent probe to estimate mitochondrial membrane potential by flow cytometry failed to show any difference between groups although this technique allows detection of small change in membrane potential induced by respiratory chain inhibitors or uncoupling agents (Fig. 3).

Analysis of mitochondrial membrane composition showed a slight effect linked to diabetes progression but exhibited some differences between rat genotypes. For lean rats, length of fatty acid presented in phospholipids was decreased with the age, whereas poly‐unsaturation and percentage of w‐3 were increased. The opposite was observed for ZDF fa/fa rats, as chain length was increased and unsaturation level tended to decrease with the age (Table 3).

**Table 3:** Liver mitochondrial membrane fluidity and specifications

	11 weeks		14 weeks	
	lean	fa/fa	lean	fa/fa
5 NS (polar head)×10^−3^	575 ± 4^a^	580 ± 8^a^	560 ± 9^b^	570 ± 4^a^
16 NS (hydrophobic part)×10^−3^	767 ± 7^a^	866 ± 11^b^	828 ± 9^b^	843 ± 24^b^
Chain length (A.U.)	7.59 ± 0.25^a^	5.33 ± 0.52^b^	6.21 ± 0.14^b,c^	7.10 ± 0.30^a,c^
Unsaturation (A.U.)	7.08 ± 0.69	8.89 ± 1.05	8.01 ± 0.20	7.80 ± 0.39
Poly unsaturation (A.U.)	1.68 ± 0.17^a^	2.20 ± 0.25^b^	2.04 ± 0.03	1.94 ± 0.08
% w3	2.70 ± 0.23^a^	3.28 ± 0.22^b^	3.50 ± 0.11^b^	3.79 ± 0.09^b^

The membrane fluidity was estimated using ESR experiments and membranes specifications by NMR. Data are presented as mean ± SEM (*n* = 7). *a* ≠ *b* with *P* < 0.05 on each line. a and b are statistically different.

Membrane rigidity closed to polar head estimated with 5 NS marker, deceased with the age in lean animal while it remained unchanged in ZDF fa/fa rats. Membrane fluidity observed on hydrophobic part, with 16 NS marker, was increased with age in ZDF lean and it was maintained in diabetic rats (Table 3).

Otherwise, protein levels of mitochondrial respiratory complex (CI‐IV), ATP synthase and UCP2 content were not altered with diabetes but decreased with aging (Table 4 and Figure S2). However, ANT2 content was lower in ZDF fa/fa rats regardless of the age.

**Table 4:** Content of mitochondrial respiratory chain complex in ZDF rats liver mitochondria

	11 weeks		14 weeks	
	lean	fa/fa	lean	fa/fa
Complex I	1.00 ± 0.10	0.91 ± 0.07	1.02 ± 0.12	1.04 ± 0.22
Complex II	1.00 ± 0.07	1.03 ± 0.06	1.09 ± 0.13	1.06 ± 0.23
Complex III	1.00 ± 0.03	1.12 ± 0.29	1.07 ± 0.17	1.32 ± 0.24
Complex IV	1.00 ± 0.09	0.97 ± 0.18	1.02 ± 0.13	1.16 ± 0.21
ATP synthase	1.00 ± 0.11	1.04 ± 0.06	1.08 ± 0.14	1.02 ± 0.12
ANT2	1.00 ± 0.03^a^	0.78 ± 0.04^b^	0.94 ± 0.06^a^	0.61 ± 0.04^b^
UCP2	1.00 ± 0.05^a^	1.14 ± 0.06^a^	0.66 ± 0.07^b^	0.65 ± 0.03^b^

Protein expressions of complex I‐IV, ATP synthase, ANT2, UCP2 were determined by Western blotting. Data were normalized to the expression of VDAC‐1 and are presented as mean ± SEM (*n* = 7). *a* ≠ *b* with *P* < 0.05 on each line. a and b are statistically different.

### Mitochondrial calcium retention capacity

As ROS production and membrane fluidity affect mitochondrial PTP opening, we investigated calcium retention capacity (CRC), another important mitochondrial function involved in cell death and insulin signaling (Taddeo et al. 2014). CRC (corresponding to the amount of Ca^2+^ required to induce permeability transition) was measured by loading mitochondria with a train of Ca^2+^ pulses until a fast Ca^2+^ release occurred, which marks the onset of the permeability transition. Basal CRC was reduced in ZDF fa/fa 14w rats compared to their 11w littermates indicating increased vulnerability to PTP opening. This was significant with GM as substrates (−20%), but less evident with succinate (−14%). As shown in Table 5 and in the literature (Batandier et al. 2006), Cyclosporine A (CsA) enhanced CRC of about 100% in the presence of G/M and about 70% with succinate in liver mitochondria (Table 5).

**Table 5:** Effect of CsA on the Ca^2+^ retention capacity of isolated ZDF rats mitochondria

	11 weeks		14 weeks	
	lean	fa/fa	lean	fa/fa
G/M (*μ*molCa^2+^ mg Prot^−1^)	235 ± 13	275 ± 18^a^	233 ± 14	219 ± 19^b^
G/M + CsA (*μ*molCa^2+^ mg Prot^−1^)	515 ± 19^a^	445 ± 28	525 ± 35^a^	417 ± 35^b^
CsA‐G/M (*μ*molCa^2+^ mg Prot^−1^)	280 ± 10^a^	170 ± 17^b^	293 ± 25^a^	198 ± 20^b^
Succ (*μ*molCa^2+^ mg Prot^−1^)	225 ± 18^a^	290 ± 6^b^	210 ± 15^a^	250 ± 20
Succ + CsA (*μ*molCa^2+^ mg Prot^−1^)	380 ± 12	400 ± 8^a^	348 ± 20^b^	363 ± 15
CsA‐Succ (*μ*molCa^2+^ mg Prot^−1^)	155 ± 18^a^	110 ± 6^b^	138 ± 18	112 ± 12^b^

The CRC were determined on liver mitochondria extracted from ZDF fa/fa or lean rats at the age of 11 weeks or 14 weeks using glutamate/malate (G/M) or succinate (Succ) as substrates and either in the presence or not of CsA. Effect of CsA was calculated as the difference between CRC in the presence or not of 1 μmol L^−1^ CsA. Data are presented as mean ± SEM (*n* = 7). *a* ≠ *b* with *P* < 0.05 on each line. a and b are statistically different.

**Figure 4:**
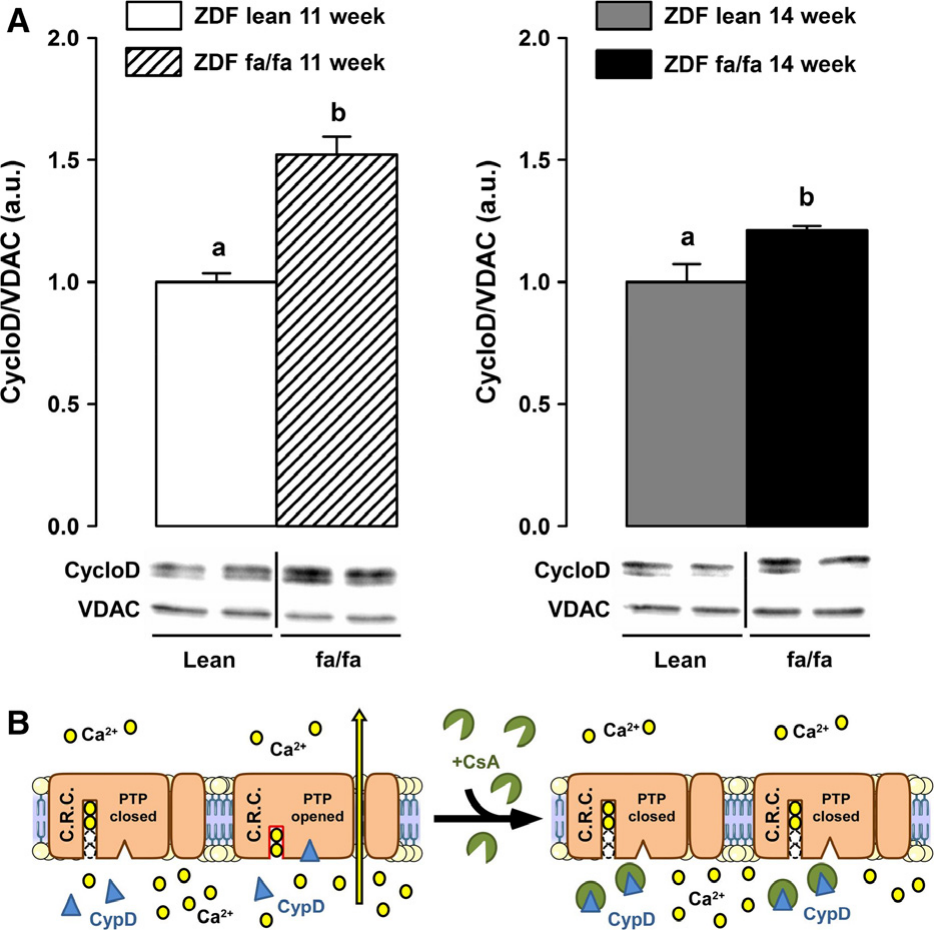
ZDF fa/fa or lean mitochondrial membrane attached cyclophilin D content. (A) Liver mitochondria were isolated as described in Materials and Methods. Mitochondria were lysed and membrane collected before proteins extraction. CyclophilinD contents were determined by western blot and normalized to the expression of VDAC‐1. Values are expressed as means ± SEM (*n* = 7 rats in each group). *a* ≠ *b* with *P* < 0.05 on each graph.(B) Schematic representation of PTP opening regulation.

In ZDF lean rats, CRC in either the absence or presence of CsA were unchanged with the age. In diabetic animals, CRC decreased with the development of the disease. Nevertheless, the effect of CsA was not altered with aging but significantly decreased according to genotype. In fact, at 14‐weeks‐old, its effect on CRC in the presence of G/M was 32% less in ZDF fa/fa mitochondria than in their own control (Table 5). As CsA acts through binding to the cyclophilin D protein (CypD), that regulates or constitutes part of the mitochondrial permeability transition pore (PTP), we looked at possible changes in CypD concentration and localization in ZDF rats. Estimation of mitochondrial membrane attached CypD by Western blot revealed that it was higher in diabetic animals. Depending on the age (11‐weeks‐old vs. 14‐weeks‐old), it was 50% or 21% higher in ZDF fa/fa rats (Fig. 4).

## Discussion

In ZDF rat, a model of progressive development of T2D, characterized by insulin resistance and hyperglycemia, we analyzed whether the establishment of the disease was correlated with changes in liver mitochondrial function. Mitochondrial function was assessed in ZDF fa/fa rats at the age of 11 and 14 weeks as well as in normoglycemic – normoinsulinemic littermate control rats (ZDF lean fa/+). First, we observed no changes in mitochondrial respiration with G/M or Succinate between diabetic or nondiabetic rats. Surprisingly, palmitoyl‐carnitine driven mitochondrial respiration increases with the age in lean animals but not in diabetic rats, suggesting that the use of fatty acids could be impaired during the first weeks of the development of diabetes. Moreover, we reported an increase with age of the maximal uncoupled respiration with TMPD/Ascorbate in ZDF lean but not in diabetic animals, suggesting a lack of adaptation of the respiration in the latter. The main diabetes‐related difference in oxidative phosphorylation was the reduced RCR observed in 14 weeks‐old ZDF fa/fa rats. This partly supports Ramsey et al.'s observation showing diabetes‐induced mitochondrial uncoupling. Our study supports the idea that a minimal duration of hyperglycemia is necessary before achieving that dysfunction. The phenomenon is associated neither with an increased UCP2 content nor an augmented ROS release. It can only be explained by the observed increased basal vulnerability of PTP opening noticed in the ZDF fa/fa 14 w rats (decrease CRC) and maybe to an altered matrix calcium homeostasis. Mitochondrial respiration was associated with ROS release but was not related linearly to the respiratory flux. Therefore, we analyzed H_2_O_2_ production in different conditions despite the lack of modification in respiration rate. In the specific conditions established to performed reverse electron flux through complex I (and by calculating the difference between H_2_O_2_ production with succinate alone and with succinate + rotenone), we surprisingly found that H_2_O_2_ release by the respiratory chain was lower in ZDF fa/fa 14w rats as compared to ZDF fa/fa 11w and to ZDF lean 11 and 14w (102 vs. 157; 146 and 123 pmol min^−1^ mg^−1^, respectively). This observation suggests that the development of diabetes seems to be concomitant to a decreased ROS production induced by reverse electron flux through complex I (*i.e.,* from CII to CI). The decrease in ROS release is still reinforced by an elevated GPx activity in ZDF fa/fa rats which could contribute to decrease ROS damages. These adaptations seem to prevent oxidative stress in ZDF fa/fa rats, as GSSG and aconitase/fumarase were not so much different than lean rats at the same age. To further characterize the function of liver mitochondria, we analyzed mitochondrial membrane potential and CRC. TMRM fluorescence analysis showed no differences between groups suggesting that mitochondrial potential is not altered during the development of diabetes in this rat model. On the other hand, CRC was impaired in the sense that CsA appeared to be less effective in inhibiting the PTP opening in ZDF fa/fa at the age of 14 weeks, independently of the substrate used. The lower inhibitory effect of CsA could be due to the higher membrane‐bound content of CypD, as CsA acts through inhibiting CypD in PTP opening at this location (Fig. 4B) (Nicolli et al. 1996). However, this assumption is paradoxical since it has been demonstrated that the more CypD is attached to the membrane, the more the CRC is decreased and conversely. Finally, because the binding sites of CypD on PTP and on other proteins remains unknown, a more credible hypothesis could be a decrease in the affinity of CypD to PTP‐binding sites associated with an increase for other partners in ZDF fa/fa mitochondria. Elucidating the origin of the differences in membrane‐bound CypD content in ZDF fa/fa rats compared to ZDF lean needs further investigations.

Considering that the mitochondrial function, especially PTP opening, is tightly controlled by respiratory chain complexes content and their lipid environment, we analyzed mitochondrial membrane composition and fluidity in ZDF rats. No change in respiratory complex proteins was observed and thus these contents could not be responsible for change in mitochondrial activity. Moreover, the dynamics of the mitochondrial membrane was studied at different levels. The order parameters 5 NS and 16 NS, assessed membrane viscosity at the polar head or at the hydrophilic portion, respectively (Gornicki and Gutsze 2000). As shown in Table 3, the 5 NS and 16 NS markers were elevated in diabetic groups so that the fluidity was decreased in ZDF fa/fa rats. These observations could be due to an increase in fatty acids chain length. Surprisingly, Zucker diabetic rats presented only slight changes in unsaturation or poly‐unsaturation.

In conclusion, this study shows that liver mitochondrial dysfunction is not strongly associated with the establishment of diabetes in this insulin‐resistant rat model. After 3 or 6 weeks with hyperglycemia, liver mitochondrial parameters remain broadly unchanged, contrary to what was observed in the muscle (Lenaers et al. 2010). Interestingly enough, we showed subtle modifications in PTP regulation that could take place during maturation and adaptive response to hyperglycemia and hyperinsulinemia. These results are in agreement with studies showing links between PTP opening control, CypD content, and insulin resistance (Taddeo et al. 2014; Tubbs et al. 2014) that should now be taken into consideration to develop new therapeutic targets.

## Conflicts of Interest

None declared.

## Supporting information




**Figure S1.** Liver histology : Histological changes in the ZDF rat liver tissues were evaluated using H&E staining.

**Figure S2.** Representative western‐blots presented in Table 4.
