# Supplementary figures and images for: Liver mitochondrial function in ZDF rats during the early stages of diabetes disease
Source: Physiol Rep. 2016 Feb 4;4(3):e12686. doi: 10.14814/phy2.12686 (PMC4758924; doi:10.14814/phy2.12686)

ZDF lean

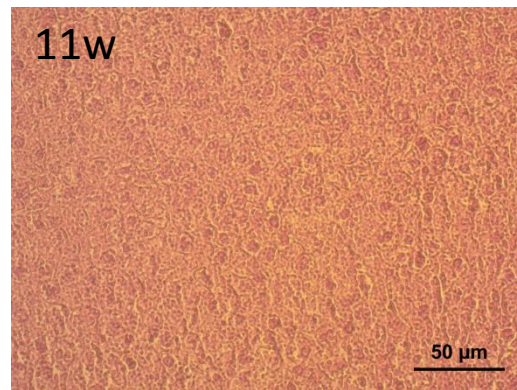

ZDF fa/fa

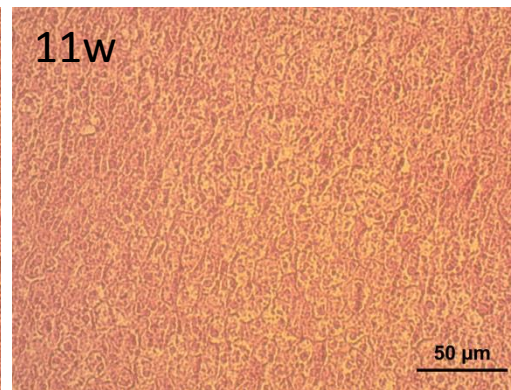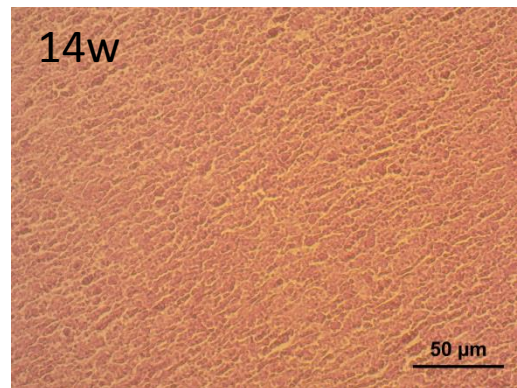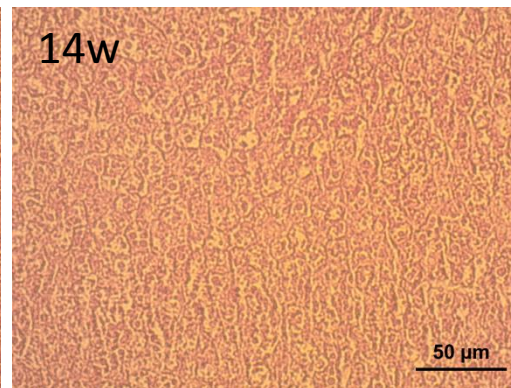

Supplement: Supplementary file 1 — Figure S1. Liver histology : Histological changes in the ZDF rat liver tissues were evaluated using H&E staining. [file PHY2-4-e12686-s001.pdf]

## Supplementary figure 1

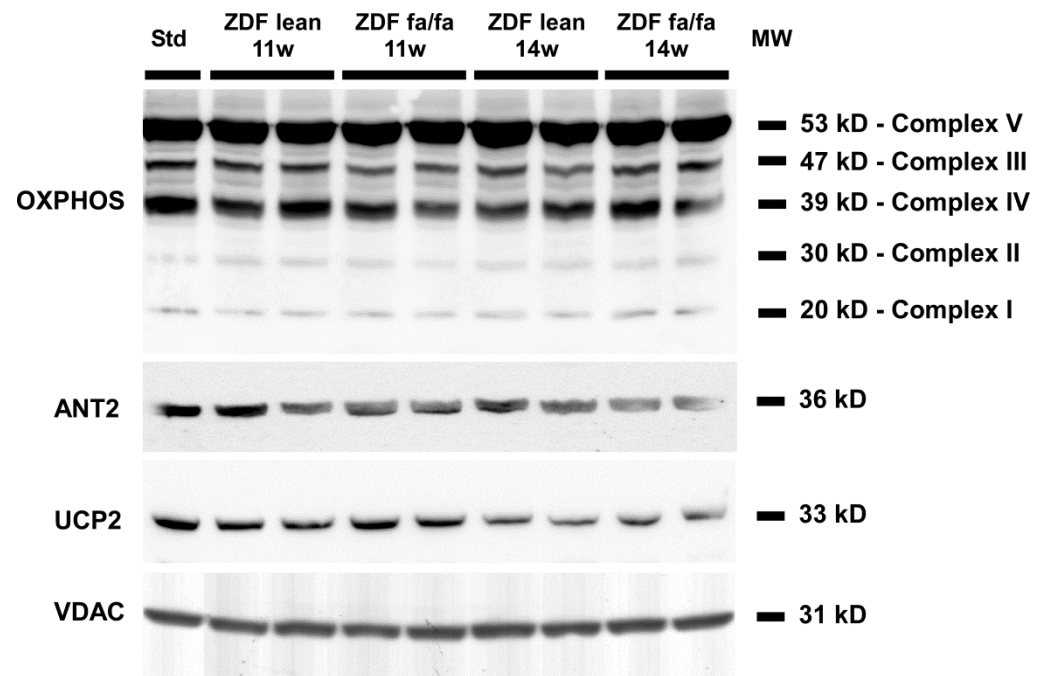

Supplement: Supplementary file 2 — Figure S2. Representative western‐blots presented in Table 4. [file PHY2-4-e12686-s002.pdf]
